# A case series of patients with filamin-C truncating variants attending a specialized cardiac genetic clinic

**DOI:** 10.1093/ehjcr/ytad572

**Published:** 2023-11-17

**Authors:** Sophie Hespe, Julia C Isbister, Johan Duflou, Raj Puranik, Richard D Bagnall, Christopher Semsarian, Belinda Gray, Jodie Ingles

**Affiliations:** Genomics and Inherited Disease Program, Garvan Institute of Medical Research, and University of New South Wales, 384 Victoria Street, Darlinghurst, 2010 NSW, Australia; Faculty of Medicine and Health, The University of Sydney, Camperdown, 2050 NSW, Australia; Faculty of Medicine and Health, The University of Sydney, Camperdown, 2050 NSW, Australia; Department of Cardiology, Royal Prince Alfred Hospital, Camperdown, 2050 NSW, Australia; Agnes Ginges Centre for Molecular Cardiology at Centenary Institute, The University of Sydney, Sydney, 2050 NSW, Australia; Faculty of Medicine and Health, The University of Sydney, Camperdown, 2050 NSW, Australia; Faculty of Medicine and Health, The University of Sydney, Camperdown, 2050 NSW, Australia; Department of Cardiology, Royal Prince Alfred Hospital, Camperdown, 2050 NSW, Australia; Faculty of Medicine and Health, The University of Sydney, Camperdown, 2050 NSW, Australia; Agnes Ginges Centre for Molecular Cardiology at Centenary Institute, The University of Sydney, Sydney, 2050 NSW, Australia; Faculty of Medicine and Health, The University of Sydney, Camperdown, 2050 NSW, Australia; Department of Cardiology, Royal Prince Alfred Hospital, Camperdown, 2050 NSW, Australia; Agnes Ginges Centre for Molecular Cardiology at Centenary Institute, The University of Sydney, Sydney, 2050 NSW, Australia; Faculty of Medicine and Health, The University of Sydney, Camperdown, 2050 NSW, Australia; Department of Cardiology, Royal Prince Alfred Hospital, Camperdown, 2050 NSW, Australia; Genomics and Inherited Disease Program, Garvan Institute of Medical Research, and University of New South Wales, 384 Victoria Street, Darlinghurst, 2010 NSW, Australia; Faculty of Medicine and Health, The University of Sydney, Camperdown, 2050 NSW, Australia; Department of Cardiology, Royal Prince Alfred Hospital, Camperdown, 2050 NSW, Australia

**Keywords:** Filamin-C, Genetic testing, Arrhythmogenic cardiomyopathy, Sudden cardiac death, Case series

## Abstract

**Background:**

*FLNC* encodes for filamin-C, a protein expressed in Z-discs of cardiac and skeletal muscle, involved in intracellular signalling and mechanical stabilization. Variants can cause diverse phenotypes with skeletal (myofibrillar or distal myopathy) and/or cardiac (hypertrophic, restrictive, and arrhythmogenic cardiomyopathies) manifestations. Truncating variants have recently been implicated in arrhythmogenic cardiomyopathy (ACM) without skeletal disease.

**Case summary:**

Retrospective review of medical records, including cardiac investigations, was performed for families attending a specialized clinic with a *FLNC* truncating variant (*FLNC*tv). Variants were classified according to accepted variant interpretation criteria. Of seven families identified, six had primary cardiac phenotypes with one nonsense and five frameshift variants (nonsense-mediated decay competent) identified. One family had no cardiac phenotype, with a pathogenic variant (p.Arg2467Alafs*62) identified as secondary genetic finding. Of the six with cardiac phenotypes, proband age at diagnosis ranged 27–35 years (four females). Five families experienced sudden cardiac death (SCD) of a young relative (age range: 30–43 years), and one patient listed for cardiac transplant. Left ventricular (LV) ejection fraction ranged from 13 to 46%, with LV fibrosis (late gadolinium enhancement) on cardiac imaging or on postmortem histology seen in three families. Two families had one genotype-positive/phenotype-negative relative.

**Discussion:**

The *FLNC*tv causes a left-sided ACM phenotype with a high risk of severe cardiac outcomes including end-stage heart failure and SCD. Incomplete penetrance is observed with implications for reporting secondary genetic findings.

**Learning points**

- *FLNC* truncating variants cause a distinct and severe cardiac phenotype, characterized by a left-sided arrhythmogenic cardiomyopathy phenotype with high risk of severe cardiac outcomes including end-stage heart failure and sudden cardiac death.
- *FLNC* truncating presents an exciting example of the insights that can be gleaned from better understanding genotype-specific disease subgroups, with benefit for patient management and outcomes.

## Introduction

**Figure 1:**
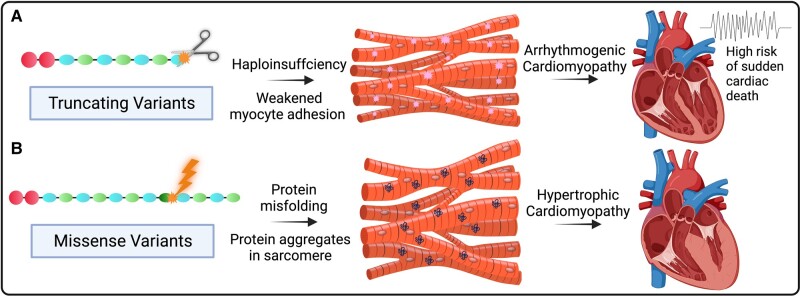
Mechanism of *FLNC* variants. Graphic of understood pathomechanism of variant types. (*A*) Truncating variants causing arrhythmogenic cardiomyopathy, while (*B*) missense variants cause hypertrophic and restrictive cardiomyopathies.^3,4^ Created with BioRender.com.

*FLNC* encodes for filamin-C, a protein expressed in Z-discs of cardiac and skeletal muscle, involved in intracellular signalling and mechanical stabilization.^1^ Variants in *FLNC* can cause diverse phenotypes with skeletal (myofibrillar or distal myopathy) and/or cardiac (hypertrophic, restrictive, dilated, and arrhythmogenic cardiomyopathies) manifestations that are inherited in an autosomal dominant pattern. Missense variants, which interfere with dimerization and folding of the protein lead to protein aggregates in the sarcomere, have been associated with hypertrophic and/or restrictive cardiomyopathy phenotypes (*Figure 1*).^2,3^*FLNC* presents as an example in cardiac genetics of the value of better characterizing genotype-specific disease subgroups. Evidence to support genotype-guided recommendations now exist in disease guidelines, specifically stating those with an *FLNC* truncating variant (*FLNC*tv) and left ventricular ejection fraction (LVEF) < 50% are at increased risk of ventricular arrhythmia and consideration of an implantable cardioverter defibrillator (ICD) is warranted.^4^

*FLNC* is highly constrained for loss-of-function variation,^5^ with *FLNC*tv that are predicted to undergo nonsense-mediated decay and result in haploinsufficiency recently implicated in arrhythmogenic cardiomyopathy (ACM) and dilated cardiomyopathy (DCM) with LV fibrosis and ventricular arrhythmia,^1,2,6^ due to weakening of the myocyte adhesion. We present our experience of managing families with *FLNC*tv in a specialized clinic in Sydney, Australia.

## Methods

Those attending a specialized multidisciplinary genetic heart disease clinic at the Royal Prince Alfred Hospital, who had a likely pathogenic or pathogenic truncating variant in *FLNC*, and provided written informed consent were included. Approximately 1700 unrelated patients have been evaluated in this clinic, including genetic testing, since 2002. This included at-risk relatives attending for clinical screening where the proband was deceased and postmortem genetic testing had been performed. We reviewed medical records, including clinical history, family history, and genetic test reports. Cardiac investigations included echocardiogram, electrocardiogram (ECG), and cardiac magnetic resonance (CMR) imaging. Genetic variants from both research and clinical genetic reports were reviewed, and those with putative loss-of-function variants, i.e. truncating variants such as insertions or deletions leading to a frameshift or nonsense variants, were included. Variants were classified according to the American College of Medical Genetics and Genomics (ACMG) and Association of Molecular Pathologists standards for variant interpretation.^7^ Approval was granted by Sydney Local Health District Human Research Ethics Committee, and participants, or their next of kin, provided written informed consent.

**Figure 2:**
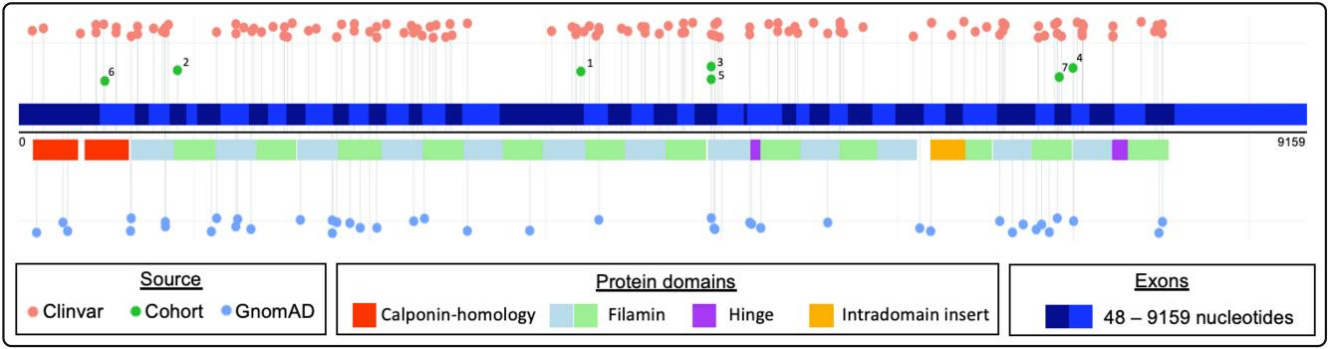
Gene topology of *FLNC* truncating variants (cases and controls). Gene topology of *FLNC* above the line shows exons starting with Exon 1 at nucleotide position 0, defined in Ensembl transcript: ENST00000325888.13; below the line are types of protein domains, defined in UniProt Q14315 FLNC_HUMAN; and reported truncating variants are shown as coloured dots, shown above the line in cases [our cohort (green and numbered) and ClinVar reports (red)] and below the line in controls (blue, gnomADv2.1).

Patients are summarized in *Table 1*. The *FLNC*tv in ClinVar (*n* = 120; date: August 2022) and gnomAD v2.1 (*n* = 42; date: August 2022) were extracted and plotted on a gene topology figure along with our case variants (*n* = 7; *Figure 2*). Exons were defined by Ensembl transcript ENST00000325888.13 and protein domains by UniProt Q14315 FLNC_HUMAN.

**Table 1:** Summary table of the clinical, genetic, and family history of each case

Case	1	2	3	4	5	6	7
Variant	p.Ala1334fs*	p.Ala380fs*	p.Val1643fs*	p.Gln2499fs*	p.Val1643fs*	p.Trp206*	p.Arg2467Alafs*
Classification	Likely pathogenic	Likely pathogenic	Pathogenic	Pathogenic	Pathogenic	Likely pathogenic	Likely pathogenic
Age of onset	38	27	29	35	35	31	NA
Deceased	Yes (SCD)	No	No	No	Yes (SCD)	No	No
Type of imaging	PM	Echo	CMR	CMR	PM	Echo	Echo
Ventricular arrhythmia	Yes (SCD)	Yes (VT)	No	Yes (PVC burden)	Yes (SCD)	Yes (OHCA)	No
Fibro-fatty infiltration	Yes (PM)	No	Yes (CMR-LGE)	No	Yes (PM)	No	No
LV dilation	Yes	Yes	Yes	Yes	Yes	No	No
Reduced systolic function	—	Yes	Yes	Yes	—	Yes	No
Family history	SCD (first-degree relative)	DCM and SCD (father)	DCM and SCD (mother), DCM (brother)	Mild DCM (mother)	No	SCD (father)	No
Variant in family members	No testing	No testing	No testing	No testing	1 Sibling	Father (SCD), 1 sibling	Father
Number of G+/P− individuals in family	—	—	—	—	1	1	2

Variant classifications are from ACMG criteria PVS1 and PM2 for likely pathogenic variants, with the addition of PS4_M for the pathogenic variants.

SCD, sudden cardiac death; PM, postmortem; CMR, cardiac magnetic resonance; Echo, echocardiography; OHCA, out-of-hospital cardiac arrest; VT, ventricular tachycardia; PVC, premature ventricular contraction; CMR-LGE, cardiac magnetic resonance late gadolinium enhancement; DCM, dilated cardiomyopathy; G+/P−, genotype positive/phenotype negative.

### Patient 1

Patient 1 suffered a sudden cardiac death (SCD) aged 38 years while attending to her child. An initial postmortem examination was performed by a forensic pathologist, and cause of death was determined to be unascertained and due to possible cardiac arrhythmia. Macroscopically, the heart showed no abnormalities with the anterior LV wall measuring 13 mm, lateral 10 mm, and posterior 9 mm in thickness. The interventricular septum measured 11 mm in thickness and the anterior right ventricular (RV) wall 4 mm. Microscopically, sections from the RV and LV showed no significant abnormalities. Postmortem exome sequencing was performed in a research laboratory and identified a likely pathogenic deletion resulting in a frameshift and a premature stop codon in *FLNC* c.4000_4015del, p.Ala1334Profs*6, and a missense variant of uncertain significance in *KCNH2* c.2274G>T, p.Gly925Val. The *FLNC* variant is absent in gnomAD and has not been reported in literature. Postmortem cardiac histology sections were re-examined by an expert forensic pathologist blinded to the genetic result, who identified areas of fibrosis with fatty infiltration within the LV free wall and septum and a limited chronic inflammatory cell infiltrate in a subendocardial band type distribution in some sections. Possible healing of LV myocardial infarction was noted in a single focus area shown by advanced tissue granulation. There was general hypertrophy of the LV myocytes but with no disarray or unusual nuclear morphology and minimal changes in the RV. Expert cardiac pathologist review concluded that this was a possible case of LV-dominant ACM. The family history revealed a first-degree relative who died suddenly aged 42 years with postmortem evaluation reporting the cause of death as pulmonary oedema but no additional information is available.

### Patient 2

Patient 2 presented during pregnancy at 27 years of age. She was asymptomatic and underwent cardiac assessment after her father suffered a SCD at 43 years playing cricket, 7 years after a diagnosis of DCM. Initial assessment showed impaired LV function and non-sustained ventricular tachycardia (NSVT) on Holter monitoring. She developed episodes of recurrent VT leading to a caesarean section at 33 weeks and subsequently underwent insertion of an ICD. She experienced complications with an ICD lead failure, which required lead extraction. During her second pregnancy, there were further complications from the extraction with superior vena cava (SVC) obstruction, requiring venoplasty initially leading to a hospital admission at 26 weeks of pregnancy until delivery at 32 weeks, followed by SVC repair and reconstruction. She later developed systolic dysfunction, with an LVEF of 15%, and was stabilized on medical therapy. At 40 years of age, she developed worsening heart failure [New York Heart Association (NYHA) Class III and IV symptoms], leading to an acute hospital admission for inotropic support. Her repeat echocardiogram showed deterioration in LV function (LVEF 30%), with heart failure medications up-titrated and consideration for cardiac transplant. She responded well to medical therapy, stabilizing with improved systolic function (LVEF 42%), NYHA status (Class II), and one run of NSVT over the following 3 years. Research-based exome sequencing identified a likely pathogenic deletion resulting in a frameshift and a premature stop codon in *FLNC*, c.1134_1135delGT, p.Ala380Profs*19. The variant is absent in gnomAD and has not been reported in literature.

### Patient 3

**Figure 3:**
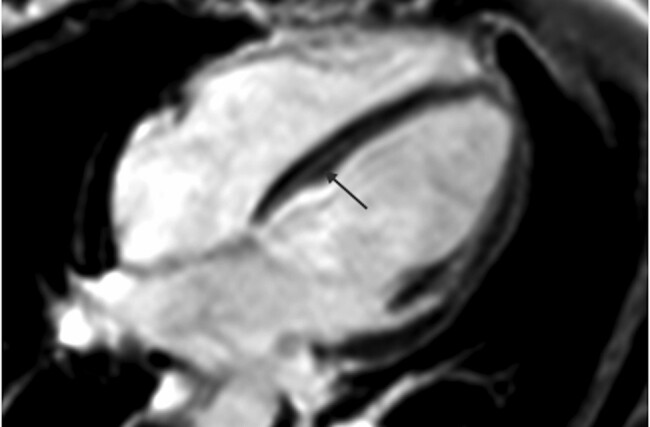
Cardiac magnetic resonance imaging of Case 3. A four-chamber view with contrast showing late gadolinium enhancement of the midwall from basal to midseptum as see by arrow.

Patient 3 presented for clinical screening at 29 years due to strong family history of DCM. Initial cardiac assessment revealed normal sinus rhythm with mild ST depression in the lateral leads on ECG. Her echocardiogram showed cardiac dimensions at the upper limit of normal: LV end-diastolic dimension indexed (LVEDDi) 32.7 mm/m^2^, LV end-systolic diameter (LVESD) 23.5 mm/m^2^, and low normal systolic function (LVEF 52%). Over the next 8 years, she remained asymptomatic with serial imaging demonstrating mildly dilated LV with mildly reduced systolic function (LVEF ∼46%), leading to a diagnosis of DCM. At 38 years of age, she underwent CMR imaging demonstrating normal LV size with mild–moderate impairment of systolic function (LVEF 45%), normal RV size with mild impairment of systolic function (RVEF 49%), and typical basal–midseptal midwall late gadolinium enhancement (LGE, as seen by arrown in *Figure 3*).^8^ Research-based exome sequencing identified a pathogenic insertion resulting in a frameshift and a premature stop codon in *FLNC*, *c.4926_4927insACGTCACA*, p.Val1643Thrfs*26. The variant has been reported twice in ClinVar in cases with cardiomyopathy and seen once in gnomAD. There is a positive family history of SCD with her mother dying in her early fifties with DCM and her brother dying at age 26 years from progressive heart failure and DCM, but DNA is not available for cascade genetic testing.

### Patient 4

**Figure 4:**
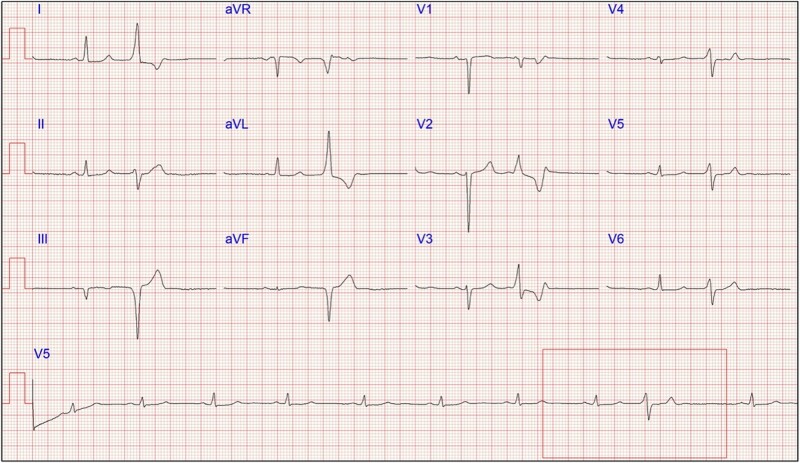
Electrocardiogram of Case 4. Captured premature ventricular contraction (square box) showing a superior axis in V1 and a tall broad R-wave in V2 and a positive axis laterally, placing the origin at the basal–midinferior left ventricle towards the septum.

Patient 4 presented with general lethargy at 35 years of age and was referred for cardiac investigations. Twenty-four-hour Holter monitor showed significant burden of ∼10 000 multifocal ventricular ectopic beats (11% total beats) and 12-lead ECG demonstrating the origin of the predominant PVC to be basal–midinferior LV (*Figure 4*). Cardiac magnetic resonance imaging demonstrated a severely dilated LV (LVEDVi 132 mL/m^2^) with mildly reduced systolic function (LVEF 46%), as well as mildly dilated RV [RV end-diastolic volume indexed (RVEDVi) 120 mL/m^2^] with low normal systolic function (RVEF 51%) and no LGE. Research-based exome sequencing identified a pathogenic insertion resulting in a frameshift and a premature stop codon in *FLNC*, c.7496_7497insTGCT, p.Gln2499Hisfs*46. The variant has been reported once in ClinVar and is absent in gnomAD. The patient’s mother has a diagnosis of mild DCM, with bigeminy and couplets on a 24-h Holter monitoring. Four years after initial presentation, Patient 4 has decreased ectopic burden on Holter monitoring (∼2000 beats) and further reduction in systolic function (LVEF 38%) and dilation (LVEDVi 136 mL/m^2^).

### Patient 5

**Figure 5:**
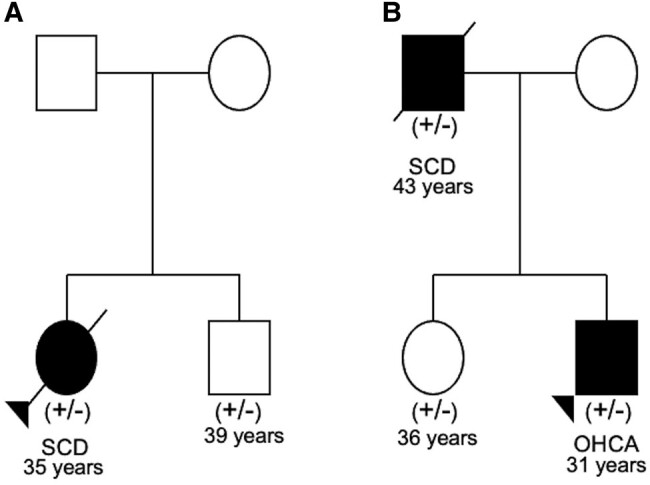
Family pedigrees. (*A*) Case 5 with variant *FLNC* c.4926_4927insACGTCACA, p.Val1643Thrfs*26. (*B*) Case 6 with variant *FLNC* c.617G>A p.Trp206Ter. SCD, sudden cardiac death; OHCA, out-of-hospital cardiac arrest.

Patient 5 died of SCD aged 35 years at work. Postmortem examination revealed moderate LV dilation (45 mm), limited small foci of myocardial replacement fibrosis in the LV free wall, and a minor degree of non-transmural fatty infiltration of the outflow tract. The postmortem investigation concluded that these changes may be DCM, albeit without significant enlargement and no evidence of congestive heart failure. Postmortem exome sequencing identified the same variant as Case 3, a pathogenic insertion resulting in a frameshift and premature stop codon in *FLNC* c.4926_4927insACGTCACA, p.Val1643Thrfs*26. The patient’s asymptomatic sibling underwent cascade genetic testing and was found to carry the same variant (*Figure 5A*). Comprehensive cardiac investigations showed no clear evidence of cardiomyopathy; however, CMR imaging showed a limited focus of LGE on the RV side of the basal septum.

### Patient 6

Patient 6 presented at 31 years following an out-of-hospital cardiac arrest. Prolonged cardiopulmonary resuscitation (60–70 min), 11 automatic external defibrillator shocks, and 6 mg of adrenaline were required to achieve return of spontaneous circulation and multiorgan dysfunction resulted. Echocardiogram revealed normal LV size and wall thickness but global impairment of LV systolic function that persisted for the duration of admission (lowest LVEF of 13%). At discharge, the principal diagnosis was ventricular fibrillation with secondary acute kidney injury, hypoxic brain injury, ischaemic bowel, ischaemic hepatitis, and anaemia. There was uncertainty regarding the underlying aetiology of the arrest and whether the LV dysfunction was the primary cause or the result of prolonged resuscitation efforts. Research-based exome sequencing identified a likely pathogenic nonsense variant resulting in a premature stop codon in *FLNC* c.617G>A p.Trp206Ter. This variant is absent in gnomAD and has not been reported previously. Family history (*Figure 5B*) identified the patient’s father suffered a SCD at 43 years, with cause determined to be myocarditis at postmortem. Analysis of postmortem DNA showed he also carried the *FLNC*tv. Further, cascade genetic testing of the patient’s sister identified she carries the familial variant, though all cardiac investigations are within normal parameters at age of 35 years.

### Patient 7

Patient 7 presented at 17 years of age with delayed development and syndromic features consistent with Noonan’s syndrome. Clinical trio whole exome sequencing was performed, and a pathogenic *de novo* variant explaining the clinical diagnosis was identified. In addition, a paternally inherited likely pathogenic deletion resulting in a frameshift and a premature stop codon in *FLNC*, c.7399del, p.Arg2467Alafs*62, was reported. The variant is absent in gnomAD and has not been reported in literature. Subsequent cardiac investigations were performed in both the patient and her father, and all showed normal cardiac parameters. Ongoing clinical screening every 12 months was advised until the patient reaches 20 years old, then every 2–3 years if investigations remain unremarkable.

## Discussion

We report a series of patients attending a specialized cardiac genetic clinic with a putative loss-of-function *FLNC*tv. The phenotype is characterized by left-sided ACM in the presence of fibro-fatty infiltration of the myocardium with high risk of severe cardiac outcomes including end-stage heart failure and SCD.^1^ This phenotype is congruent with the understood pathomechanism of *FLNC*tv, i.e. truncation of the protein, leading to putative loss of function of the allele causing haploinsufficiency (*Figure 1*). Filamin-C crosslinks with actin filaments at the Z-disc between sarcomeres. Insufficient production of filamin-C leads to weakened myocyte adhesion at the molecular level, resulting in thinning and dilation of the myocardium and loss of myocytes with fibro-fatty replacement.^6^ In comparison, in-frame deletions and missense variants likely cause misfolding of filamin-C, with deposition of large protein aggregates within the sarcomere at the Z-disc, resulting in larger fibre diameter of the myocardium and an HCM or RCM phenotype.^3^

Of the seven families identified, six had primary cardiac phenotypes, with proband age at diagnosis ranging from 27 to 35 years and 4/6 females. Four families experienced SCD of a young relative (age range: 30–43 years). Cardiac imaging was available for five affected individuals across four families, with LV dilation and systolic dysfunction reported in all (LVEF range: 13–46%). Left ventricular fibro-fatty infiltration of the myocardium, demonstrated as LGE on CMR (*Figure 3*) or on postmortem histology, was seen in three families. For all families, there is marked variability in clinical presentation, from ventricular arrhythmias to end-stage heart failure, as well as examples of non-penetrance.

While existing literature and examples from this case series support *FLNC*tv as a cause of ACM with high risk of ventricular arrhythmia and heart failure, increasing reports highlight these variants identified as secondary genetic findings, i.e. identified in individuals without the ACM phenotype undergoing genetic testing for another purpose.^9,10^ However, these cases may not necessarily be benign bystanders, with carriers in the general population potentially at increased risk of ventricular arrhythmia.^10^ Non-penetrance was seen in Family 7, where the *FLNC*tv was identified secondary to another identified genetic cause explaining their non-cardiac condition. Further, at-risk family members who had undergone cascade genetic testing in Families 5 and 6 demonstrate additional examples of non-penetrance (*Figure 5*), with those individuals having normal cardiac investigations, including one male with limited focus of septal LGE on CMR imaging. In the large population reference database, gnomAD, truncating variants occur infrequently, with each being individually rare (*Figure 2*), demonstrating intolerance of *FLNC* to loss-of-function variation (pLI = 1 and LOEUF = 0.25).^5^ Cases, both from ClinVar and our case series, show causative variants distributed throughout the gene suggesting gene-wide constraint. Existing literature reports high penetrance of disease (97% at >40 years), and guidelines recommend early ICD implantation in high-risk affected patients.^1^ In addition, it is broadly recommended that these families are managed in specialist multidisciplinary clinics including genetic counselling, given the nuances regarding clinical heterogeneity and incomplete penetrance.

## Conclusion

*FLNC* truncating variants cause a distinct and severe cardiac phenotype, characterized by a left-sided ACM phenotype with a high risk of severe cardiac outcomes including end-stage heart failure and SCD. The phenotype is underpinned by a pathomechanism specific to this variant type. Non-penetrance exists and highlights that other factors modulating disease expression remain incompletely understood. The *FLNC*tv presents an exciting example of the insights that can be gleaned from better understanding genotype-specific disease subgroups, with benefit for patient management and outcomes.

## Data Availability

The data underlying this article will be shared upon reasonable request to the corresponding author.

## Lead author biography

Sophie Hespe has a background as a cardiac physiologist and is currently a student under supervision of A/Prof Jodie Ingles in the Clinical Genomic Laboratory at Garvan Institute of Medical Research, Sydney, Australia.

**Consent:** All patients, mentioned relatives, and next of kin of deceased patients have provided written informed consent for this study in compliance with COPE guidelines.

**Funding:** B.G. is the recipient of a National Health and Medical Research Council (NHMRC) Early Career Fellowship (#1122330). R.D.B. is supported by a New South Wales Health Cardiovascular Disease Senior Scientist Grant. C.S. is the recipient of a NHMRC Practitioner Fellowship (#1154992) and a New South Wales Health Cardiovascular Disease Clinician Scientist Grant. J.I. is the recipient of an National Health and Medical Research Council (NHMRC) Career Development Fellowship (#1162929). This work was supported in part by a NSW Health Cardiovascular Health EMCR grant.
